# Historical changes in the distribution of the Sichuan golden snub‐nosed monkey (*Rhinopithecus roxellana*) in Sichuan Province, China

**DOI:** 10.1002/ece3.11270

**Published:** 2024-04-16

**Authors:** Yunchuan Dai, Wancai Xia, Yujing Zhu, Charlotte Hacker, Xueyu Wang, Dayong Li

**Affiliations:** ^1^ Key Laboratory of Southwest China Wildlife Resources Conservation (Ministry of Education) China West Normal University Nanchong Sichuan Province China; ^2^ Institute for Ecology and Environmental Resources Chongqing Academy of Social Sciences Chongqing China; ^3^ Key Laboratory of Conservation Biology of *Rhinopithecus roxellana* at China West Normal University of Sichuan Province Nanchong Sichuan Province China; ^4^ U.S. Fish and Wildlife Service Falls Church Virginia USA

**Keywords:** conservation, distribution changes, human activities, *Rhinopithecus roxellana*

## Abstract

The Sichuan golden snub‐nosed monkey (*Rhinopithecus roxellana*) is a rare and endangered primate species endemic to China. Conducting research on the population distribution changes of the Sichuan golden snub‐nosed monkey holds paramount importance for its conservation. Our study represented a comprehensive investigation into the population distribution of the Sichuan snub‐nosed monkey by integrating data acquired from field surveys, protected areas, and historical records and using Geographic Information Systems (GIS) to explore changes in distribution across various time periods, including the historical (the Mid‐to‐Late Pleistocene), recent (1980–2000), and current (2001–2023). The research findings demonstrate a significant shift in the distribution range of the Sichuan golden snub‐nosed monkey compared to historical time frames. Notably, between 1980 and 2000, there was a sharp decline in distribution area. Analyses revealed that the southernmost distribution county for the Sichuan golden snub‐nosed monkey in Sichuan Province has shifted northward from Huili to Kangding. Furthermore, distribution changes in Sichuan Province are not solely characterized by a reduction in habitat area but also by a decrease in vertical distribution zones. Regions in the northeastern part of Sichuan with elevations below 1000 m, such as Guang'an City, Bazhong City, Dazhou City, and Nanchong City, no longer support the presence of the Sichuan golden snub‐nosed monkey. At present, the distribution range is confined to elevations between 1000 and 4000 m in the two major mountain ranges of Qionglai and Minshan. A holistic approach is required to safeguard this species. The establishment of movement corridors can play a critical role in enhancing the overall connectivity of current distribution areas. Additionally, we propose implementing a hierarchical approach to protect current habitats. Spatially differentiated conservation measures should be implemented to prioritize the protection of key habitats while simultaneously monitoring anthropogenic activities in non‐key habitats to prevent further fragmentation and isolation of the monkey's distribution areas.

## Graphical abstract

Historical changes in the distribution of the Sichuan golden snub‐nosed monkey were identified. Between 1980 and 2000, there was a sharp decline in the distribution area of the Sichuan golden snub‐nosed monkey. The boundary of the distribution area for the Sichuan golden snub‐nosed monkeys in Sichuan Province has significantly shifted northward. Distribution changes of Sichuan golden snub‐nosed monkeys are not solely characterized by a reduction in habitat area but also by a decrease in vertical distribution zones.

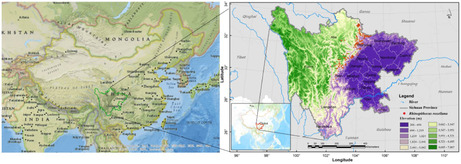

## 1 INTRODUCTION

Habitat reduction and fragmentation have emerged as critical factors contributing to the endangerment and potential extinction of species. Since the 1970s, there has been an estimated 25% decline in vertebrate animal populations (Dirzo et al., 2014). Non‐human primates play a large role in maintaining biodiversity; however, alarming statistics indicate that more than half of non‐human primate species are currently at risk of extinction (Carvalho et al., 2019; Estrada et al., 2017; Zhang et al., 2019). The golden snub‐nosed monkey is a taxonomic group within the genus *Rhinopithecus*, consisting of five distinct species: the Yunnan snub‐nosed monkey (*Rhinopithecus bieti*), gray snub‐nosed monkey (*R. brelichi*), Sichuan golden snub‐nosed monkey (*R. roxellana*), Myanmar snub‐nosed monkey (*R. strykeri*), and Tonkin snub‐nosed monkey (*R. avunculus*; Xiang, 2020). The first four species, including the Sichuan golden snub‐nosed monkey, are indigenous to China and hold the official status of national first‐level protected animals.

Golden snub‐nosed monkeys once inhabited a vast territory across China, spanning North, East, Central‐South, Southwest, Northwest, and Northeast China (Wen, 1995). While their presence in East China was extensive, the scarcity of documentation presents challenges in accurately assessing their historical distribution. The Qinling‐Huaihe Line serves as the geographical boundary separating southern and northern China, delineating the northern boundary of the evergreen broad‐leaved forest, the preferred habitat for the Sichuan snub‐nosed monkey. Consequently, this line significantly influences primate distribution, with the majority of their populations concentrated south of it (Zhang, 2002). Currently, snub‐nosed monkeys persist in fragmented and isolated populations within the four regions of East China, Central‐South China, Southwest China, and Northwest China, gradually diminishing in numbers eastward. In northwest China, the golden snub‐nosed monkey is exclusively distributed in Shaanxi and Gansu provinces. The distribution pattern of snub‐nosed monkeys reflects the influence of climate and vegetation zoning on animal distribution within China's mainland monsoon region. Overall, these primates exhibit transitional characteristics, including a southward shift of the northern boundary, a northward shift of the southern boundary, and a westward shift of the eastern boundary (Liu, 2007).

Current understanding suggests that the decline in snub‐nosed monkey distribution can be attributed primarily to three factors: the species' behavioral characteristics, the natural environment, and human activities (Liu, 2007). Snub‐nosed monkeys possess specific dietary requirements, such as a preference for high‐altitude vegetation, seasonal fruits, and a reliance on lichens. These dietary preferences play a crucial role in shaping their foraging behavior and habitat selection. They exhibit relatively weak survival abilities, limited evasion and defense mechanisms against predators, and lower reproductive rates (Li et al., 2009; Yang et al., 2009). These factors contribute to their limited adaptability in challenging environments. Climate change inevitably induces changes in microclimates within their habitats, consequently impacting the environment and behavior of snub‐nosed monkeys. Research has revealed that, in response to climate change, various species, including snub‐nosed monkeys, have undergone shifts in their geographic distribution patterns, such as moving towards higher altitudes (McCarty, 2001; Root et al., 2003; Walther et al., 2002; Wuethrich, 2000).

Human activities also play a substantial role in the reduction of the snub‐nosed monkey's distribution range. Human activities have progressively intensified in China's western region, leading to an escalated exploitation of natural resources and an unprecedented rate of habitat destruction (Huang et al., 2021; Zhao et al., 2019). Increased demands on the environment, including the cultivation of crops, urban development, and the expansion of transportation networks, exert a profound impact on the overall integrity of the monkey's habitat. Moreover, deliberate afforestation by humans has resulted in changes to the dominant tree species within the monkey's habitat. These alterations in vegetation composition may render the habitat less suitable for these species, potentially triggering migration behavior (Wen, 1995). Collectively, these multifaceted factors are likely the primary drivers contributing to the distribution reduction of the snub‐nosed monkey. Historically, the Sichuan golden snub‐nosed monkey once inhabited extensive areas in southern, southwestern, central, and northwestern China (Li et al., 2002). Fossil records indicate that its distribution was considerably broader during the Early and Middle Pleistocene epochs compared to the present (Jablonski, 1998; Pan & Jablonski, 1987; Pan & Oxnard, 2001). Despite the implementation of a series of protective measures by the Chinese government, yielding significant achievements, the scientific preservation of the golden snub‐nosed monkey remains an urgent and unresolved question. Since 1989, Sichuan Province in China has undertaken a series of prioritized forestry development projects. However, the impact of climate change and human activities, including widespread deforestation, agricultural expansion, hunting, logging, and habitat destruction, has persistently diminished the geographic range of the golden snub‐nosed monkey population (Zhao et al., 2018, 2019).

Our study represents a comprehensive investigation into the population distribution of the Sichuan snub‐nosed monkey by integrating data acquired from field surveys, protected areas, and historical records. The research encompasses three distinct periods: historical (the Mid‐to‐Late Pleistocene), recent (1980–2000), and current distribution (2001–2023). By incorporating reference materials with a broader temporal range, a more nuanced understanding of the changes in the Sichuan golden snub‐nosed monkey's distribution range can be achieved. The analysis of the species' distribution across different time periods yields invaluable insights into its adaptive responses to environmental changes and human activities. It serves as a crucial benchmark for evaluating the extent of habitat loss and fragmentation, enabling the identification of critical areas that require targeted conservation interventions.

## 2 MATERIALS AND METHODS

### 2.1 Study area

**FIGURE 1:**
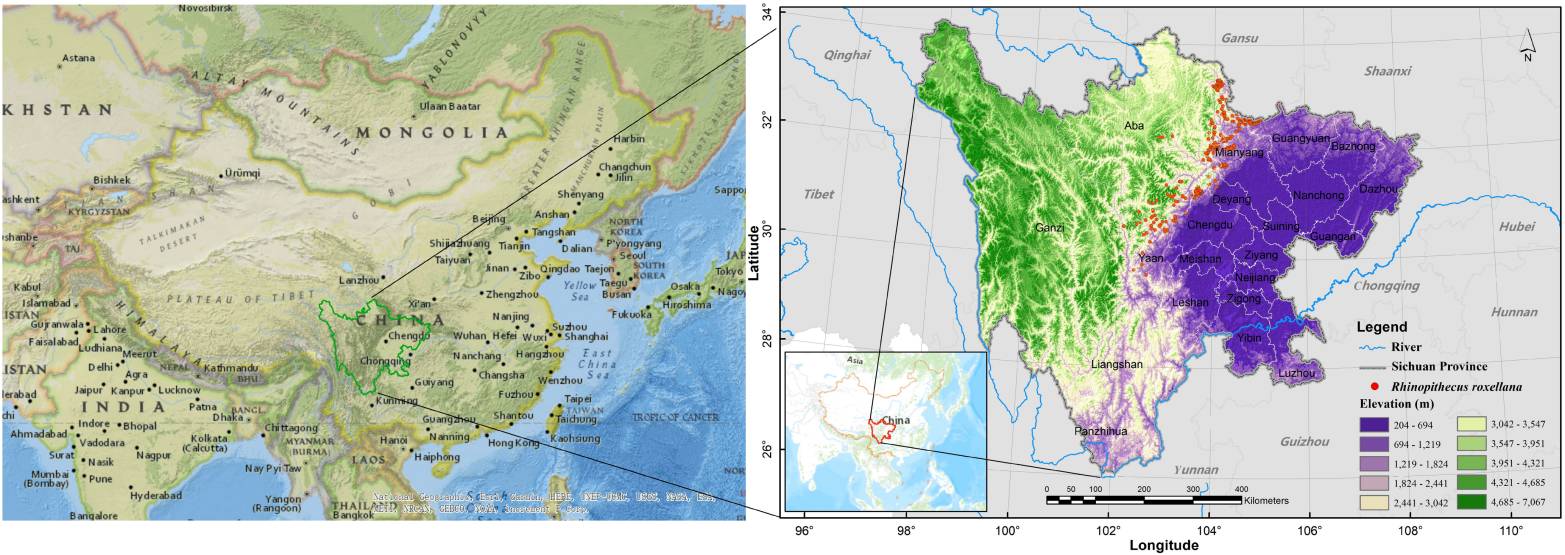
The location of study area and the current occurrences of the Sichuan golden snub‐nosed monkey in Sichuan Province, China.

Sichuan Province is in the southwestern portion of China, positioned upstream of the Yangtze River, and spans approximately 1000 km from east to west and 900 km from north to south (Figure 1). It shares borders with Chongqing Municipality to the east, Yunnan and Guizhou Provinces to the south, Tibet Autonomous Region to the west, and Qinghai, Gansu, and Shaanxi Provinces to the north. With a land area of 480,000 km^2^, Sichuan Province ranks fifth in size among all provinces in China. The mountains of Southwest China are renowned for their remarkable genetic diversity of terrestrial vertebrates and are geographically positioned between the Qinghai‐Tibet Plateau and the plains along the middle and lower reaches of the Yangtze River (Mi et al., 2021). The western part of Sichuan Province exhibits higher elevations compared to the eastern part. Sichuan Province boasts abundant animal and plant resources, representing a significant biological gene bank globally. It holds the distinction of having the highest number of terrestrial wild animal species among all provinces in China, encompassing over 1000 vertebrate species (Department of Ecology and Environment of Sichuan Province; http://sthjt.sc.gov.cn/). This includes more than 200 mammal species, over 600 bird species, over 80 reptile species, over 90 amphibian species, and over 200 fish species (Department of Ecology and Environment of Sichuan Province; http://sthjt.sc.gov.cn/). Mammals and birds alone account for over half of the total species in China. As of 2018, Sichuan Province had established a network of 129 nature reserves, covering a combined area of 7.3461 million km^2^. These reserves accounted for 88.51% of the total nature reserve area within the province (Department of Ecology and Environment of Sichuan Province; http://sthjt.sc.gov.cn/). Among these protected areas, 29 have been identified as habitats for the Sichuan golden snub‐nosed monkey (Wen, 2009).

### 2.2 Data sources

Our study was based on data collected from field surveys conducted by our research team, complemented by information derived from protected areas and documented records, to investigate the historical (Mid‐to‐Late Pleistocene), recent (1980–2000), and current (2001–2023; Appendix S1) distribution range of the Sichuan golden snub‐nosed monkey. During the review of older reference materials (Table 1), we encountered various terms such as “狨” (rónɡ), “猴” (hóu), “狖” (shù), and “禺” (yú), which were used to refer to the Sichuan golden snub‐nosed monkey. We selected corresponding location information to represent the distribution of the snub‐nosed monkey. However, due to the significant time span involved, verifying the accuracy of some older data poses challenges, and as a result, we adopted a passive acceptance approach, assuming the information to be factual. Historical distribution information includes fossil sites dating back to the Mid‐to‐Late Pleistocene period as well as ancient textual records spanning various periods in Chinese history. By utilizing reference materials with a broader time span, we aim to achieve a more comprehensive understanding of the changes in the distribution range, providing valuable insights into the true dynamics of the distribution area. It is imperative to highlight that historical data corresponding to past periods have been deliberately excluded from Appendix S1. The accurate determination of historical geographical locations, utilizing precise latitude and longitude coordinates, poses considerable challenges. Consequently, the historical data incorporated in this study are predominantly confined to township‐level regions, as opposed to being precisely delineated with specific latitude and longitude positions.

**TABLE 1:** Distribution data of the Sichuan golden snub‐nosed monkey at different periods in Sichuan Province, China.

	Data sources				
	Published literature	Historical documents	Chorography	Field survey	Other record
Historical distribution	Wen (1995), Qin et al. (1999), Quan and Xie (2002), Wen (2009), Xiang (2020)	Classic of Mountains and Seas	/	/	Fossil Record
Distribution in 1980–2000	Wen (1995), Qin et al. (1999), Quan and Xie (2002), Zhang (2002), Wen (2009)	/	Fauna of Sichuan's Natural Resources; Records of protected areas	/	/
Distribution in 2001–2019	Liu (2007), Dirzo et al. (2014)	/	Fauna of Sichuan's Natural Resources; Records of protected areas	Population distribution survey (2010–2019)	/
2020–Present	/	/	Records of protected areas	Population distribution survey (2020–2023)	/
Species distribution points	79	32	106	387	12

### 2.3 Spatial processing of distribution information

In order to analyze the geographic distribution of the Sichuan golden snub‐nosed monkeys, we imported observation data (Appendix S1) into ArcGIS 10.6 (ESRI Inc., Redlands, CA, USA). This dataset included discrete point information where the Sichuan golden snub‐nosed monkeys were observed. Since the majority of data comprised discrete points, we used an inverse distance weighted (IDW) technique in ArcGIS 10.6 to create a heat map, enabling a more intuitive understanding of the hotspots where Sichuan golden snub‐nosed monkeys are prevalent. IDW is a technique that estimates values in unknown areas based on the spatial distribution of known data points, thereby generating continuous surface data. By overlaying the species distribution points with digital elevation model (DEM) data, we obtained the corresponding elevations of the species distribution points, thus gaining insights into the elevation distribution of the species. The DEM was extracted from the ASTER GDEM V2 DEM with a resolution of 30 m (http://www.gscloud.cn/).

### 2.4 Habitat variation

Given the challenges in evaluating habitat quality data for Sichuan golden snub‐nosed monkeys across different historical periods, we endeavored to utilize forest changes as an indicator of habitat alteration. This involved assessing the relative quantity and spatial changes of the local forest area. We consulted various sources, including local chronicles (Qin et al., 1999), forestry records, forest bureau, archives from the Sichuan Provincial Museum, and data on the distribution and logging volumes of timber yards and commercial timber during historical periods (Appendix S2). Our study integrates two primary datasets. Firstly, we collected primary survey data in the field conducted by our research team. Secondly, we gathered historical distribution data from published articles, local chronicles, and archival records. While historical data points may not be precisely mapped to coordinates, they are reliably situated at the township level, representing one of the most trustworthy methods for acquiring historical data. Therefore, we express confidence in the reliability of the data we possess. To investigate the altitudinal gradient changes in the distribution of Sichuan golden snub‐nosed monkeys across different temporal periods, we conducted analyses by overlaying the collected data points onto the DEM of Sichuan Province. Altitude information was extracted from these data points to examine the trends in the altitudinal distribution of Sichuan golden snub‐nosed monkeys across various historical periods.

## 3 RESULTS

### 3.1 Distribution of the Sichuan golden snub‐nosed monkey at present

**FIGURE 2:**
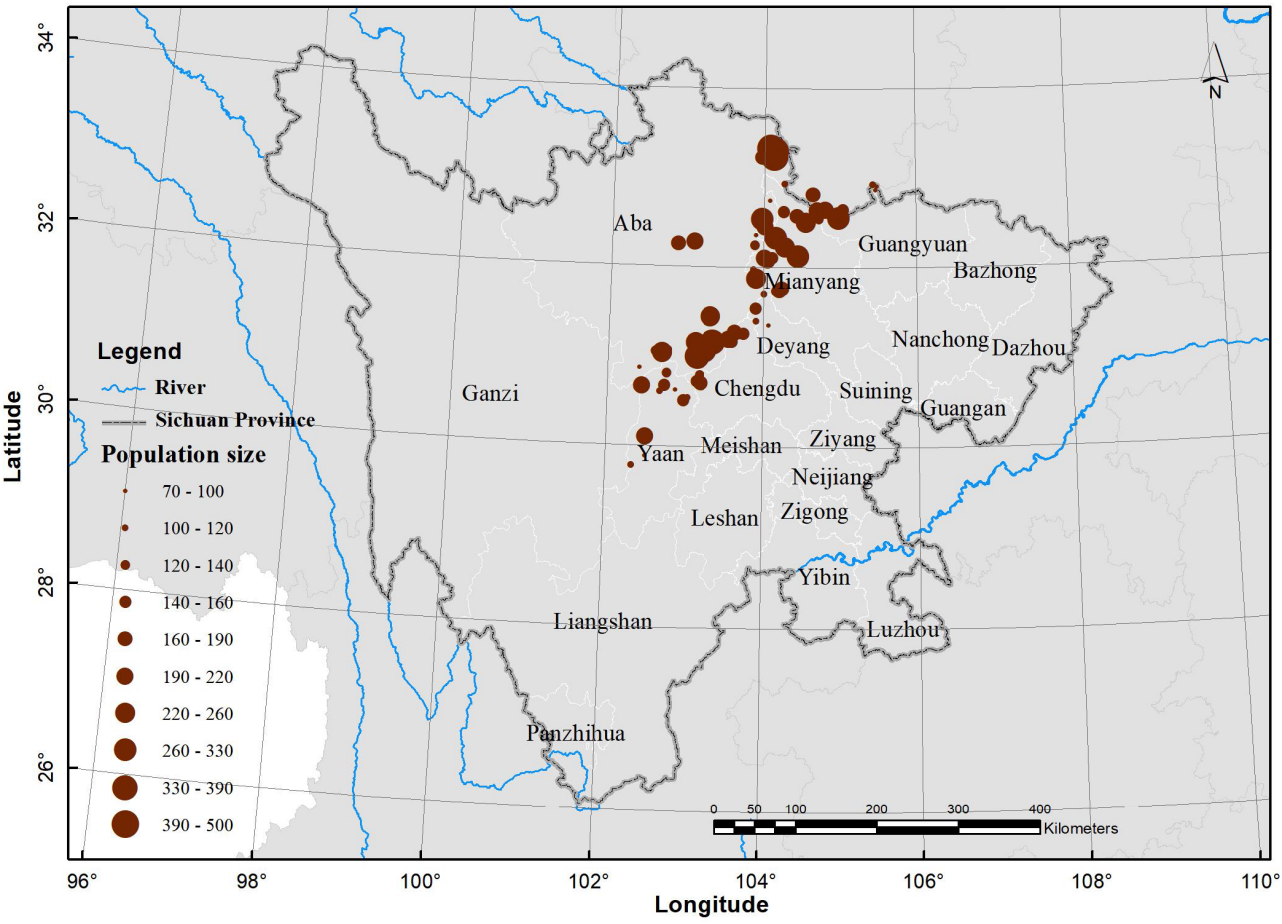
Distribution of the Sichuan golden snub‐nosed monkey at present in Sichuan Province, China.

**FIGURE 3:**
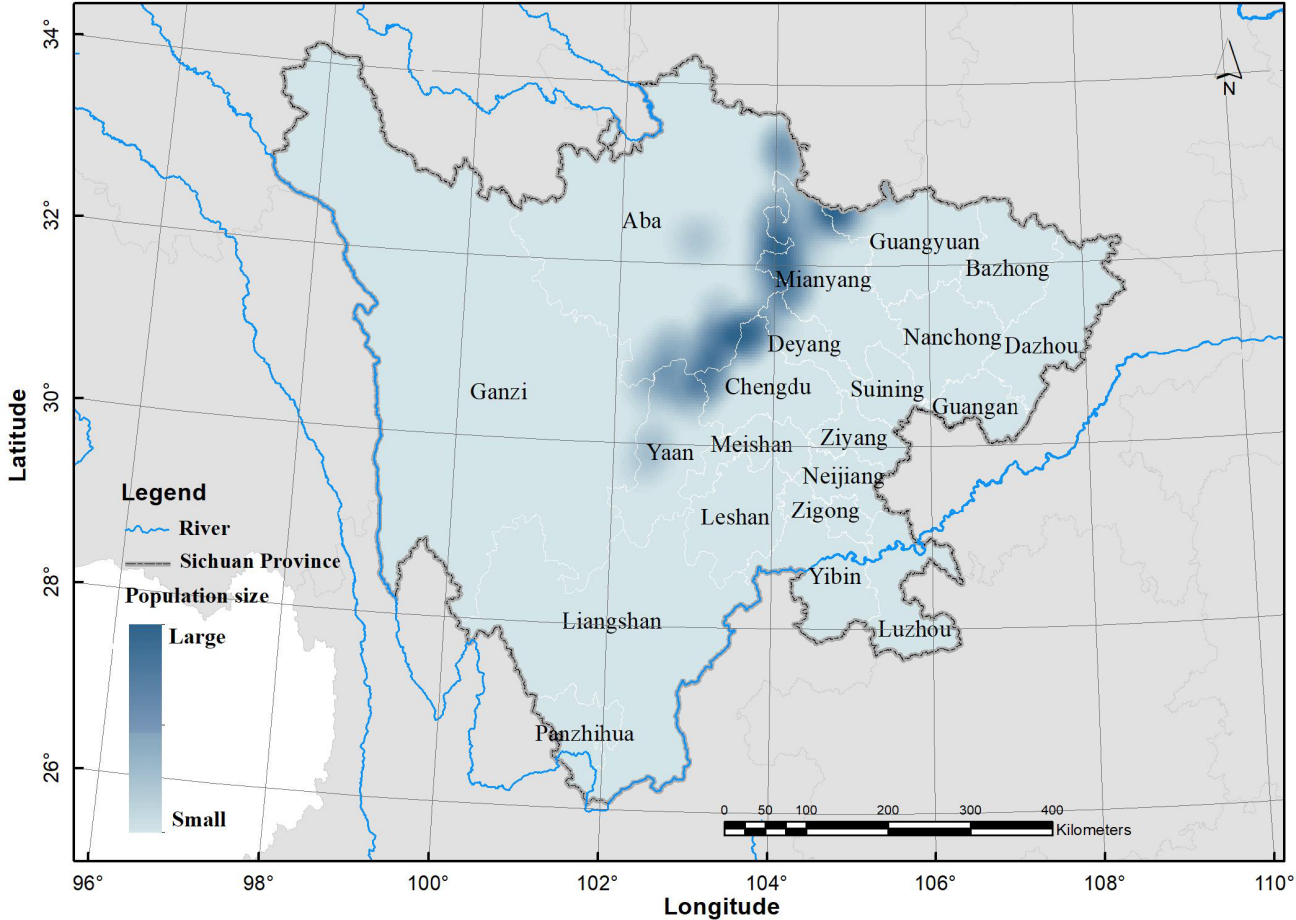
Population size (an IDW interpolation) of the Sichuan golden snub‐nosed monkey at present in Sichuan Province, China.

According to field survey data, Sichuan golden snub‐nosed monkeys are predominantly distributed in the central and northern regions of Sichuan Province, encompassing areas such as the Aba Tibetan and Qiang Autonomous Prefecture, the northwest of Mianyang, and the northern part of Ya'an (Figure 2). Their habitat spans renowned natural reserves, including Ruoergai, Jiuzhaigou, and Wolong. The population distribution of the Sichuan golden snub‐nosed monkey is concentrated in the core protected areas of their primary locations, such as Jiuzhaigou and Wolong (Figure 3).

### 3.2 Distribution of the Sichuan golden snub‐nosed monkey at different time periods

**FIGURE 4:**
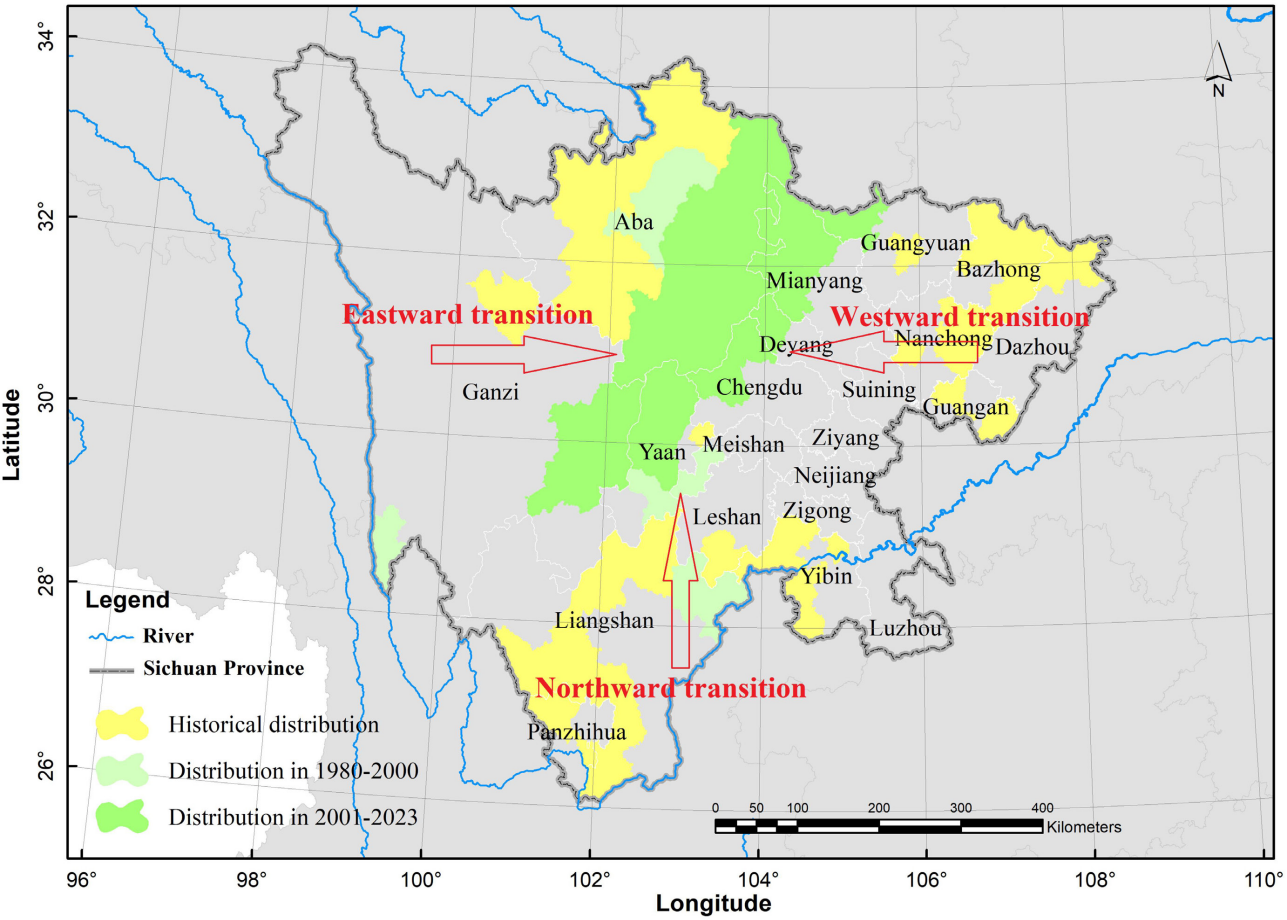
Distribution of the Sichuan golden snub‐nosed monkey at different historical periods.

Through a comparative analysis of historical records (Table 2) and field survey data, historical habitat was notably more expansive compared to its current range. Notably, between 1980 and 2000 (Figure 4), the distribution area of these monkeys experienced a drastic reduction. At present (2001–2023), the cities encompassing the southernmost distribution of Sichuan golden snub‐nosed monkeys in Sichuan Province (from east to west) are Ya'an City and Ganzi Tibetan Autonomous Prefecture. A comparison of these three time periods reveals that the species' southernmost distribution shifted northward from Huili County to Kangding. Overall, the boundary of the distribution area for the Sichuan golden snub‐nosed monkeys in Sichuan Province has significantly shifted northward (Figure 4).

**TABLE 2:** Distribution of the Sichuan golden snub‐nosed monkey at different periods in Sichuan Province, China.

Administrative region	Historical distribution	Distribution in 1980–2000	Distribution in 2001–2023
Chengdu City	Chongzhou City, Dujiangyan City, Dayi County, Pengzhou City, Pujiang County, Qionglai City	Chongzhou City, Dujiangyan City, Dayi County, Pengzhou City	Chongzhou City, Dayi County, Pengzhou City, Dujiangyan City
Deyang City	Mianzhu City, Shifang City	Mianzhu City, Shifang City	Mianzhu City, Shifang City
Ganzi Tibetan Autonomous Prefecture	Derong County, Kangding County, Luding County, Luhuo County^a^	Derong County, Kangding County, Luding County	Kangding County, Luding County
Bazhong City	Bazhong District, Nanjiang County, Pingchang County, Tongjiang County	/	/
Yibin City	Gao County, Yunlian County, Nanxi County, Pingshan County, Yibin City	/	/
Guang'an City	Guang'an District, Linshui County, Wusheng County, Yuechi County	/	/
Leshan City	Mabian Yi Autonomous County	Mabian Yi Autonomous County	
Liangshan Yi Autonomous Prefecture	Dechang County, Ganluo County, Huili County, Leibo County, Mianning County, Muli Tibetan Autonomous County, Xichang City, Yanyuan County, Yuexi County, Zhaojue County	Leibo County	/
Mianyang City	Beichuan Qiang Autonomous County, Pingwu County, An County	An County, Beichuan Qiang Autonomous County, Pingwu County	An County, Beichuan Qiang Autonomous County, Pingwu County
Aba Tibetan and Qiang Autonomous Prefecture	Aba County, Maerkang County, Heishui County, Hongyuan County, Jinchuan County, Li County, Mao County, Jiuzhaigou County, Songpan County, Wenchuan County, Xiaojin County, Ruo'ergai County	Heishui County, Hongyuan County, Li County, Mao County, Jiuzhaigou County, Songpan County, Wenchuan County, Xiaojin County	Heishui County, Li County, Mao County, Jiuzhaigou County, Songpan County, Wenchuan County, Xiaojin County
Panzhihua City	Yanbian County	/	/
Ya'an City	Baoxing County, Hanyuan County, Lushan County, Mingshan County, Tianquan County, Ya'an City, Yingjing County	Baoxing County, Hanyuan County, Lushan County, Tianquan County, Yingjing County	Baoxing County, Lushan County, Tianquan County, Yingjing County
Guangyuan City	Qingchuan County, Zhaohua District	Qingchuan County	Qingchuan County
Nanchong City	Nanchong City, Peng'an County, Xichong County, Yilong County, and Yingshan County	/	/
Dazhou City	Wanyuan City	/	/

^a^ Fossil record.

### 3.3 Changes in the average altitude of the distribution of the Sichuan golden snub‐nosed monkey

**FIGURE 5:**
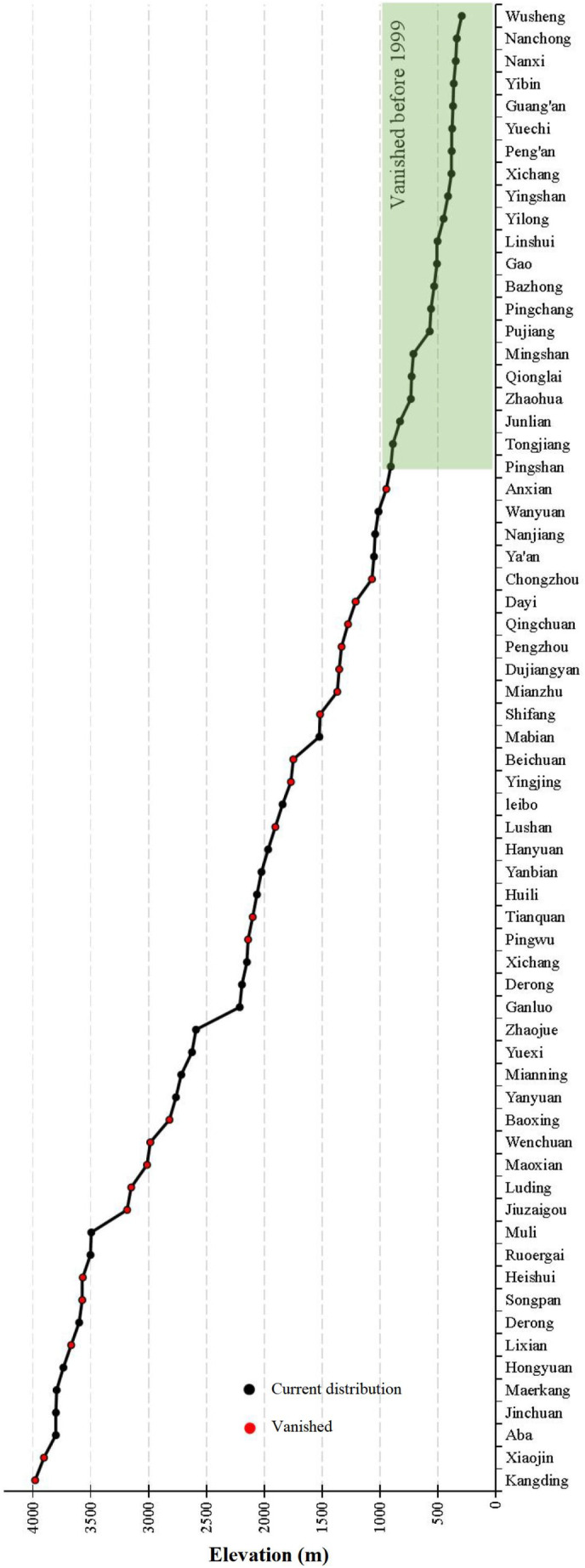
Changes in average altitude distribution of the Sichuan golden snub‐nosed monkey in Sichuan Province, China.

The reduction of the distribution of Sichuan golden snub‐nosed monkeys in Sichuan Province is not only evident in the reduction of its overall area but also in the decline of its vertical range (Figure 5). In the northeastern part of Sichuan, areas with an altitude lower than 1000 m no longer host Sichuan golden snub‐nosed monkeys (cities such as Guang'an, Dazhou, Bazhong, Nanchong, etc.). The distribution is now confined to the Qionglai and Min mountain ranges within Sichuan Province, at altitudes ranging from 1000 to 4000 m. The results indicate that between 1980 and 2000, there was a significant transformation in the distribution area of Sichuan golden snub‐nosed monkeys, with certain areas that were once part of the extensive distribution now fragmented and isolated into patches. Subsequently, these patchy distribution areas have disappeared in the current survey, leaving only a single isolated distribution area. According to historical records, the distribution area used to be a continuous expanse spanning the central part of Sichuan. Initially, the distribution of Sichuan golden snub‐nosed monkeys in the northeastern part of Sichuan vanished, followed by a decrease in their distribution in the southern part of Sichuan. Secondly, for reasons yet to be determined, the distribution area of the monkeys became fragmented, and their presence in the southern part of Sichuan is no longer observed. Finally, the once extensive distribution area became increasingly narrow, decreasing from the historical presence in 15 cities to only six cities currently (2001–2023).

### 3.4 The habitat quality of the Sichuan golden snub‐nosed monkey in different periods and human–monkey interactions

During the spring and autumn periods and the Warring States period, humans had already begun using the Sichuan golden snub‐nosed monkey habitat to some extent. However, forest resources remained relatively intact during that time, and conflicts between human activities and monkeys were not significant. In the Qin and Han dynasties, the development of iron agricultural tools in Sichuan led to rapid expansion. Alongside population growth, economic prosperity, and cultural advancements, extensive forest areas in Sichuan were transformed into farmland, resulting in a decline in the quality of the Sichuan golden snub‐nosed monkeys' habitat. During the Tang and Song dynasties, feudal society continued to prosper, and by the time of the Southern Song dynasty, the population of Sichuan reached its historical peak. Humans accelerated the utilization of forest resources, further degrading the quality of the monkeys' habitat. In the late Ming and Qing periods, the habitat of the Sichuan golden snub‐nosed monkeys became increasingly fragmented, leading to a continuous reduction in their distribution range (Table 3).

**TABLE 3:** Status and potential factors influencing habitat changes for the Sichuan golden snub‐nosed monkeys at different time periods.

Period	Year	Factors influencing the distribution changes of the Sichuan golden snub‐nosed monkeys
Spring and Autumn period and the Warring States period	770–221 BC	Humans had a certain degree of exploitation of natural resources, especially in areas like Qionglai and Lugu. They had already begun utilizing the habitat of the Sichuan golden snub‐nosed monkeys to some extent during this time, but the forest resources remained relatively intact, and conflicts between human activities and the monkeys were not significant.
The Qin and Han Dynasties	221 BC– 220 AD	Coupled with population growth, economic prosperity, and cultural advancement, extensive forest resources in Sichuan were transformed into farmland to meet the growing needs. The expansion of farmland was accompanied by a decline in forest resources. For instance, in some regions of Liangshan, forests had already started to be converted into farmland during the Han dynasty. This might be an important historical factor contributing to the reduction or even disappearance of the Sichuan golden snub‐nosed monkeys in the Liangshan area.
The Tang and Song Dynasties	618 AD–1279 AD	The population of Sichuan reached its historical peak, and human activities accelerated the utilization of forest resources, resulting in a drastic reduction of original forests in basins and hills. During this era, forest policies were lenient, allowing unrestricted deforestation for cultivation. Many hilly areas and forests in the eastern and northern parts of the Sichuan basin were converted into farmland.
The Ming and Qing Dynasties	1368–1911	Human‐induced destruction of forest resources reached its historical peak during this time. The forests in Sichuan underwent significant changes, with the primitive forests in the southern region deteriorating rapidly. In the western areas of Sichuan, evergreen broad‐leaved forests were extensively logged. In the year 1406 during the Ming Dynasty, the procurement of timber began, with wood being transported from present‐day Pingshan to Beijing for the construction of palaces. During the Qing Dynasty, in order to repay debts, the collection of forest resources was intensified. Until the end of the Qing Dynasty, in regions like Dajin, Xiaojin, and parts of Ganzi Tibetan Autonomous Prefecture in western Sichuan, extensive forests in some Tibetan‐inhabited areas turned into barren mountains due to wars.
The Republic of China	1912–1949	In 1937, the forest area in Sichuan Province was approximately 6443.4 km^2^, accounting for 13% of the total land area. About 1000 km^2^ of forests in Ebian rapidly disappeared. In the upper reaches of the Dadu River, Qingyi River, and Min River, more than 200,000 trees were felled, leading to the destruction of approximately 684 km^2^ of forest area. The upper reaches of the Dadu River include places like Yuexi, Hanyuan, and Luding. In the Min River basin, Songpan and Li counties were most significantly impacted, followed by Mao County and Wenchuan. During the Republican period, within a short period, the forest coverage rate in Sichuan decreased rapidly, and the unprecedented speed at which forest resources disappeared was astonishing.
The People's Republic of China	After 1949	Since the establishment of the People's Republic of China in 1949, the government has placed significant emphasis on wildlife conservation and has enacted a series of laws and regulations related to wildlife protection. Despite the strict conservation policies implemented by the national and provincial governments, the increasing population and rapid urbanization continue to have an impact on the distribution of the Sichuan golden snub‐nosed monkeys.

## 4 DISCUSSION

The escalation of economic activities and the surging demand for natural resources may constitute pivotal factors directly influencing the distribution pattern of the Sichuan golden snub‐nosed monkey (Li et al., 2002; Luo et al., 2015). Notably, during the recent period (1980–2000), we observed substantial changes in the distribution of the species. In the early years of the People's Republic of China foundation, driven by the imperatives of economic development, the country initiated the establishment of logging sites and conducted extensive logging activities in pristine forests (Qin et al., 1999). As the forest industry advanced, road networks gradually emerged in the prominent forest regions of Minjiang, Dadu River, and Yalong River. Moreover, a significant number of roads were constructed in proximity to smaller logging enterprises in areas like Ya'an, Mianyang, Guangyuan, and Nanchong (Qin et al., 1999). The construction and operation of these roads within forested areas unavoidably exerted an impact on the Sichuan golden snub‐nosed monkeys (Chang et al., 2012). Despite the efforts to accompany logging with afforestation during this period, the complete regeneration of forests necessitated a substantial amount of time. Furthermore, persistent human activities encircling these roads in the forested regions continued to disrupt the normal behavioral patterns of the Sichuan golden snub‐nosed monkeys. These factors have culminated in the further deterioration of their habitats, rendering some historically inhabited areas unsuitable for the continued habitation of the Sichuan golden snub‐nosed monkeys (Luo et al., 2012).

Human activities that alter various landscape structures and functions are widely observed (Brown et al., 2005; Peng et al., 2016). The study of human activities and their impact on ecosystems has consistently been a crucial focus in ecology (de la Torre et al., 2000; Quan et al., 2002). Consulting the Sichuan Yearbook reveals that during the Han Dynasty and the Western Han Dynasty, a period of relative political stability led to a substantial influx of immigrants into Sichuan Province, resulting in a population of nearly 800,000 households. The construction of roads played a pivotal role in fostering economic development, marking a period of flourishing economic and cultural growth in Sichuan's history. During the Song Dynasty, the population peaked at around 2.66 million. The intensified competition between humans and the Sichuan golden snub‐nosed monkey during this era placed immense survival pressure on the monkeys, potentially constituting a significant factor in their reduced distribution (Jiang, 1987). Research indicated that environmental and economic disparities contributed to an uneven distribution of the human Sichuan population, with population centers shifting towards the southwest of the province (Li et al., 2018). Historically, areas like Guang'an City and Nanchong City, which were once part of the distribution range of the Sichuan golden snub‐nosed monkey, have experienced a decline in their monkey populations as they transformed into population hotspots or moderately dense regions (Jiang, 1987). The Sichuan golden snub‐nosed monkey is predominantly found in areas transitioning from high to low population densities (Li et al., 2003). With a noticeable increase in livestock, roads, farmland, and other human activities, the impact of human actions on the habitat of the Sichuan golden snub‐nosed monkey has become increasingly apparent. Concurrently, wildlife exhibit strong avoidance behaviors towards livestock, roads, and farmland when selecting their habitats (Hemsworth et al., 1993). The rapid expansion of the human population is likely one of the primary reasons contributing to the shrinking distribution range of the Sichuan golden snub‐nosed monkey.

Sichuan Province, long recognized as China's prominent agricultural province with agriculture playing a pivotal role in its economic development, has undergone significant changes since 1250, when local demand for resources sharply surged, resulting in a considerable decline in forested areas. Over the centuries, farmers responded to the growing population demands by substantially increasing cultivated land through extensive land reclamation (Lee, 1982). This swift agricultural expansion had a severe impact on the habitat of the Sichuan golden snub‐nosed monkey. The pressures of logging, land reclamation, and urbanization during this period led to the fragmentation of the Sichuan golden snub‐nosed monkey's habitat, causing geographical isolation among different populations (Li et al., 2002; Zhao et al., 2018). This fragmentation not only affected different species of golden snub‐nosed monkeys but also resulted in isolation among different populations of the same species. Their habitats became fragmented into isolated small patches, posing significant challenges to their connectivity, survival, and reproduction. Concurrently, with the rapid expansion of agriculture, farmers intensified their land use, escalating conflicts with the Sichuan golden snub‐nosed monkey (Luo et al., 2016). In order to meet human demands, farmers had to further expand cultivated land or resort to forest logging. Consequently, these activities led to the loss and shrinking of the Sichuan golden snub‐nosed monkey's habitat, leaving them with inadequate refuge when confronted with disturbances from human activities (Harkness, 1998).

Despite the Chinese government's increasing implementation of strict wildlife conservation policies since the founding of the People's Republic of China in 1949, continuous population growth and urbanization still pose numerous challenges to the survival environment of the Sichuan golden snub‐nosed monkey. Our research reveals that these pressures, including logging, land reclamation, and urbanization, have resulted in the fragmentation and geographical isolation of the Sichuan golden snub‐nosed monkey populations. The observed changes in distribution over time can be directly attributed to the habitat alterations caused by swift agricultural expansion and intensified land use, as evidenced by the increased conflicts between farmers and monkeys. Despite the implementation of wildlife conservation policies, continuous population growth and urbanization pose persistent challenges, further corroborating the impact of historical and ongoing human activities on the survival environment of the Sichuan golden snub‐nosed monkey.

Apart from the impact of human activities on the distribution of wildlife, it is imperative not to overlook the influence of other natural factors on its distribution changes. Climate change, geological processes, and vegetation succession can all play significant roles in shaping their habitat (Dai et al., 2021; Dai & Li, 2023; Tarr et al., 2017). The Sichuan golden snub‐nosed monkey is typically found in higher‐altitude mountainous regions, making them susceptible to habitat shifts or expansions in response to climate variations (Grueter et al., 2009). Global warming has led to increased temperatures in high‐altitude areas, rendering their original habitats unsuitable for habitation and compelling them to migrate to higher elevations in search of suitable living conditions (Luo et al., 2015). Geological processes also impact the distribution of wildlife habitat (Zhang et al., 2011). Activities such as crustal movements and earthquakes can alter landforms, affecting the stability of their original habitats (Cheng et al., 2009). Crustal movements lead to mountain uplift or subsidence, altering the terrain and topography of mountainous regions, which subsequently influences the wildlife habitat. For instance, natural disasters like landslides and rock collapses triggered by earthquakes can damage their habitats, necessitating the search for new safe locations. Vegetation succession is another crucial natural factor affecting the wildlife habitat (DeWalt et al., 2003). Over time, vegetation undergoes changes, and alterations in different vegetation types can significantly impact their habitat selection. For example, forest degradation or shifts in vegetation types might affect the availability of food resources and alter the living environment of the Sichuan golden snub‐nosed monkey. Encouragingly, there has been a growing recognition of the imperative for conservation efforts among diverse stakeholders in recent years. This heightened awareness has prompted the development and implementation of corresponding protective management measures, such as the establishment of wildlife corridors, habitat restoration initiatives, and community‐based conservation programs. These initiatives offer promising prospects for the conservation and sustainable distribution of the Sichuan snub‐nosed monkey population.

Through the comparison of historical and contemporary distribution patterns, this study facilitates the identification of factors contributing to the reduction of the Sichuan golden snub‐nosed monkey's distribution range, thereby guiding the development of focused conservation measures. Moreover, the integration of data from protected areas and historical records significantly enhances the reliability and accuracy of the findings. This integrated approach strengthens the scientific foundation for conservation decision‐making, ensuring that protection measures effectively address the specific needs and challenges faced by the Sichuan snub‐nosed monkey. Nevertheless, the manuscript is not devoid of inherent limitations. The primary challenge lies in the inability to precisely georeference historical data to specific coordinates, thereby precluding the extraction of accurate elevation and spatial distribution information for the Sichuan golden snub‐nosed monkey during historical epochs. The historical records at our disposal, albeit invaluable, lack explicit geographic coordinates, constraining our capacity to conduct nuanced analyses of altitudinal fluctuations and spatial dynamics over temporal scales. Notwithstanding these constraints, our scholarly pursuits have entailed a rigorous scientific analysis grounded in available datasets.

## 5 CONCLUSIONS AND RECOMMENDATIONS

Compared to historical times, a significant shift in the distribution range of the Sichuan golden snub‐nosed monkey is evident in the present, particularly during the period from 1980 to 2000, which saw a sharp decline in habitat area. Through a comparison of historical records and recent and current survey data, it becomes apparent that the southernmost distribution county for the Sichuan golden snub‐nosed monkey in Sichuan Province has shifted northward from Huili to Kangding. The shifts in distribution were marked not only by a reduction in area but also by a decrease in both horizontal and vertical distribution zones for this species. Notably, areas in the northeastern part of Sichuan with elevations below 1000 m, including Guang'an City, Bazhong City, Dazhou City, and Nanchong City, no longer host the Sichuan golden snub‐nosed monkey. Presently, the distribution range of the Sichuan golden snub‐nosed monkey is confined to elevations between 1000 and 4000 m in the two major mountain ranges of Qionglai and Minshan. These findings highlight the dynamic nature of the Sichuan golden snub‐nosed monkey's habitat distribution over time, emphasizing the importance of monitoring and understanding the factors driving these changes. The observed reduction in both habitat area and vertical distribution zones raises concerns for the species' conservation, as it indicates potential challenges for their survival and population dynamics. Further research and conservation efforts are essential to safeguarding the future of this endangered primate species in the face of ongoing environmental transformations.

To further bolster conservation efforts for the Sichuan golden snub‐nosed monkey, we propose a series of comprehensive measures based on research findings. Firstly, it is imperative to establish a well‐designed top‐level conservation plan for the Sichuan golden snub‐nosed monkey. Constructing corridors between distribution areas plays a critical role in enhancing connectivity. Thus, a rational corridor planning strategy is essential to optimize habitat utilization and address the issue of a shrinking distribution range. Secondly, we advocate a hierarchical approach to protecting the monkey's habitats. Conservation efforts should be tailored according to the actual utilization patterns of the Sichuan golden snub‐nosed monkey. Key habitats must receive strict protection, while non‐key habitats necessitate enhanced human activity management to prevent excessive habitat consumption, further fragmentation, and isolation of the monkey's distribution areas. Thirdly, particular attention should be paid to the monkey's marginal distribution areas. These small, marginalized, and distant habitats, such as the regions of Jintangkongyu and Sandagu, require special attention and protection. Fourthly, the conservation of vegetation in the Sichuan golden snub‐nosed monkey's habitats should be prioritized. Mixed coniferous and broadleaf forests and shrublands are the most fragmented vegetation types and, therefore, deserve special attention. Additionally, efforts should be made to prevent further fragmentation of vegetation types with high monkey densities, such as deciduous broadleaf forests. Fifthly, the consideration of appropriate translocation measures for the Sichuan golden snub‐nosed monkey is crucial. Identifying potentially suitable habitats for the species is a key aspect, and areas with an excessive monkey population could be candidates for translocation to habitats that promote overall connectivity. Lastly, it is essential to strike a balance between development and conservation. Achieving this balance involves reconciling local economic development with the protection of the Sichuan golden snub‐nosed monkey. Effective management of human‐induced disturbances in the monkey's habitats is vital to reduce intense competition between the species and humans at different elevations. Additionally, regular climate monitoring of the monkey's habitats is indispensable for informed conservation strategies. By implementing these comprehensive measures, we can enhance the protection of the Sichuan golden snub‐nosed monkey's habitat and survival environment, thus ensuring the effective conservation and sustainable development of this endangered species.

## AUTHOR CONTRIBUTIONS

**Yunchuan Dai:** Conceptualization (lead); methodology (equal); writing – original draft (lead). **Wancai Xia:** Conceptualization (equal); writing – review and editing (equal). **Yujing Zhu:** Investigation (equal); writing – review and editing (equal). **Charlotte Hacker:** Writing – review and editing (equal). **Xueyu Wang:** Writing – review and editing (equal). **Dayong Li:** Conceptualization (lead); funding acquisition (lead); writing – review and editing (equal).

## FUNDING INFORMATION

This research was supported by the National Natural Science Foundation of China (No. 32270548), the Sichuan Science and Technology Program (2021JDRC0024), and the Second Tibetan Plateau Scientific Expedition and Research Program (No. 2019QZKK0501).

## CONFLICT OF INTEREST STATEMENT

The authors declare that they have no known competing financial interests or personal relationships that could have appeared to influence the work reported in this paper.

## Supporting information


**Appendix S1.**.

Appendix S2.

## Data Availability

The original contributions presented in the study are included in this paper; further inquiries can be directed to the corresponding author.
