# Supplementary material for: Historical changes in the distribution of the Sichuan golden snub‐nosed monkey (Rhinopithecus roxellana) in Sichuan Province, China
Source: Ecol Evol. 2024 Apr 16;14(4):e11270. doi: 10.1002/ece3.11270 (PMC11021920; doi:10.1002/ece3.11270)
Supplement: Supplementary file 2 — Appendix S2. [file ECE3-14-e11270-s001.doc]

**Appendix 2 Area of primary forest logging and its size**

| Data source | Logging areas | Area/km^2^ |
| --- | --- | --- |
| East Sichuan Logging Company | Nanchuan, Wulong, Youyang, Pengshui, Jiangbei, Guang'an, Tongliang, Zhong County, etc | 0.071 |
| South Sichuan Logging Company | Jiajiang, Emei, Ebian, Leshan, Yibin, Zigong, Gulin, Muchuan, and Pingshan | 1.524 |
| West Sichuan Logging Company | Lixian | 5.272 |
| North Sichuan Logging Company | The upper reaches of the Yangtze River, including the Fu River and the headwaters of the Jialing River. | 0.703 |
| Xikang Logging Company | Western Sichuan and eastern Tibet region | 0.283 |
| Baoxing Forestry Industry Bureau | The upper reaches of the Qingyi River, a tributary of the Min River. | 0.472 |
| Shimian Forestry Industry Bureau | Shimian forest area | 0.393 |
| Heishui Forestry Bureau | Heishui County | 6.691 |
| Ma’erkang Forestry Bureau | Ma’erkang County | 6.723 |
| Xiaojin Forestry Bureau | Xiaojin County | 3.060 |
| Guanyinqiao Forestry Bureau | Jinchuan County: Yelonggou, Taiyang River, Yishenggou, Eri River, Zhongkegou, etc. | 6.031 |
| Dajin Forestry Bureau | Jinchuan County: Dajin forest area | 2.122 |
| Longerjia Forestry Bureau | Maerkang County | 3.165 |
| Liangbei Forestry Bureau | Liangbei forest area | 4.680 |
| Longerjia Forestry Bureau | Maerkang County | 3.165 |
| Liangbei Forestry Bureau | Liangbei forest area | 4.680 |
| Kangding Forestry Bureau | Kangding City | 0.517 |
| Leibo Forestry Bureau | Leibo County, Mabian County, Pingshan County; Pingbian forestry area | 1.450 |
| Yanbian Forestry Bureau | Yanbian County | 0.011 |
| Hongqi Forestry Bureau | Wenchuan County | 0.228 |
| Nanping Forestry Bureau | Upper tributary of Bailong River, Baishui River | 1.531 |
| Luhuo Forestry Bureau | Luhuo County | 1.725 |
| Muli Forestry Bureau | Muli Tibetan Autonomous County | 1.466 |
| Daofu Forestry Bureau | Daofu, Xinlong, Qianning | 1.534 |
| Xinlong Forestry Bureau | Xinlong County | 1.647 |
| Maoergai Forestry Bureau | Songpan County | 1.491 |
| Songpan Forestry Bureau | Songpan County | 1.249 |
| Jiajinshan Forestry Bureau | Baoxing County | 0.095 |
| Li Qiuhe Forestry Bureau | Kangding County | 0.263 |
